# Pre-Migration Trauma Exposure and Mental Health Functioning among Central American Migrants Arriving at the US Border

**DOI:** 10.1371/journal.pone.0168692

**Published:** 2017-01-10

**Authors:** Allen Keller, Amy Joscelyne, Megan Granski, Barry Rosenfeld

**Affiliations:** 1 Program for Survivors of Torture, Bellevue Hospital/New York University School of Medicine, New York, NY, United States of America; 2 Department of Psychology, Fordham University, Bronx, NY, United States of America; Queensland University of Technology, AUSTRALIA

## Abstract

In recent years, increasing numbers of families and individuals have arrived at the U.S. border from Central America, in particular, from Honduras, El Salvador, and Guatemala. This study sought to examine pre-migration trauma exposure and current mental health functioning of migrant families arriving at the U.S. border from the Northern Triangle region, with specific attention to the reasons offered for leaving their home country and the frequency with which migrant families appear to satisfy legal criteria for asylum We interviewed 234 adults in McAllen, Texas, using a structured interview and standardized questionnaires to assess exposure to trauma prior to migration, reasons for leaving their home country and symptoms of posttraumatic stress and depression. We found that 191 participants (83%) cited violence as a reason for fleeing their country, 119 individuals (69%) did not report the events to the police out of fear of gang-related retaliation or police corruption, and 90% (*n* = 204) reported being afraid to return to their native country. Based on self-report symptom checklists, 32% of the sample met diagnostic criteria for PTSD (*n* = 51), 24% for depression (*n* = 36), and 17% for both disorders (*n* = 25). Examining these data against the criteria for asylum in the U.S., we found that 70% of the overall sample (*n* = 159) met criteria for asylum, including 80% of those from El Salvador, 74% from Honduras, and 41% from Guatemala. These findings suggest that the majority of Central American migrants arriving at the U.S. border have significant mental health symptoms in response to violence and persecution, and warrant careful consideration for asylum status.

## Introduction

In 2014, there was an unprecedented surge of migrants from the Northern Triangle countries (Guatemala, El Salvador, and Honduras) to the United States-Mexico border. Although public attention has focused primarily on unaccompanied minors, a comparable number of families arrived as well. In 2014 U.S. Customs and Border Patrol (CBP) apprehended roughly 50,000 unaccompanied minors in the Rio Grande Valley, versus about 20,000 in 2013, and roughly 50,000 family units, compared to only 7,000 in 2013. [1] This influx of families and children from Central America presenting at the U.S.-Mexico border was recognized by both the general public and policy makers to be an “urgent humanitarian situation,” in part because of the large number of individuals involved. [2]

Although the mass migration of Central American families has been attributed to gang violence and drug cartels, [3–5] little research has systematically examined the reasons behind this migration, or the medical and mental health of these migrants. The aim of the current study was to examine social and psychological factors contributing to this migration, in order to inform policy and decision makers regarding health and mental health of these individuals. The need for more information about Central American families arriving at the U.S. border is particularly important given that families have few legal protections. Unlike adults who arrive at the border without legal authorization to enter the U.S., families with children apprehended at the border fall under the custody of Immigrations Customs Enforcement (ICE). [6] They are typically detained and placed into expedited removal proceedings unless they express a well-founded fear of persecution if returned home, at which point they are referred for a “credible fear” hearing with an asylum officer. If deemed credible, they are referred to an immigration judge for a hearing to determine whether they are eligible for asylum. While awaiting disposition, families can be placed in a detention facility or can be released to a family member or a sponsor living in the community. [7]

In response to the dramatic increase in displaced Central American families arriving at the U.S.-Mexico border, the U.S. sharply increased its capacity to detain families as part of its “aggressive deterrence strategy.” [2] Prior to July 2014, when a 700-bed family detention facility opened in Artesia, New Mexico, ICE maintained only 100 beds for families. [8] In August 2014, ICE opened a 532-bed family detention facility in Karnes County, Texas, and another 2,400-bed facility opened in Dilley, Texas, in December 2014, replacing the Artesia, NM facility. [8] In total, these changes resulted in a 30-fold increase in the bed capacity for detained families, from 100 beds in June of 2014 to roughly 3000 beds just six months later.

The growing number of families being detained and the lack of data on the reasons for increased rates of migration is disconcerting given that prolonged detention appears to contribute substantially to the risk of depression, posttraumatic stress disorder (PTSD) and other mental health disorders in refugees and asylum seekers. [9–12] For instance, Keller et al studied asylum seekers detained in the metropolitan New York area, finding high rates of psychological distress. They identified 77% of participants with clinically significant symptoms of anxiety, 86% had depression and 50% had posttraumatic stress disorder (PTSD), and symptom severity was significantly correlated with the length of time these individuals had been detained. [9] Follow-up interviews conducted two months later revealed marked decreases in distress among those who had been released from detention (most of whom had been granted asylum) but a worsening of symptoms among those still detained. Similarly, a study of 241 refugees living in Australia found higher rates of PTSD and depression among those with longer detention experiences, suggesting that immigration detention has a negative effect on the mental health of refugees. [10]

Children and families appear particularly vulnerable to the traumatizing, unpredictable environment of immigration detention. Mares et al. interviewed families held in two Australian immigration detention facilities, observing that a lack of opportunities for education and safe play, coupled with parental distress and an undermining of the parental role, places children at a high risk for developmental psychopathology. [11] Steel et al. found precisely this outcome in ten families that had been held in an immigration detention facility in Australia for more than two years. [12] Based on interviews with 14 adults and 22 children, they found that all of the adults met diagnostic criteria for major depressive disorder and all of the children met diagnostic criteria for at least one psychiatric disorder. In short, the limited research base points to serious mental health consequences from detention for both adults and children.

The present study sought to examine pre-migration trauma exposure and current mental health functioning of migrant families arriving at the U.S. border from the Northern Triangle region. Specifically, we examined the reasons offered for leaving their home country (with specific attention to past traumatic and persecutory experiences) and the nature and severity of psychological distress (particularly depression and PTSD symptoms). In light of public debate regarding the legitimacy of this population as asylum seekers, [13–14] this study also sought to examine the frequency with which migrant families appear to satisfy legal criteria for asylum.

## Method

### Participants

Data were collected from a quasi-consecutive sample of adults seeking assistance at the Church of the Sacred Heart in McAllen, Texas. The church, located near the U.S.-Mexico border, provides shelter, food, clothing, and medical care, primarily serving women and families with children that had been apprehended by CBP and released, pending a court hearing to determine whether they had a credible fear of returning to their native country. [15] All families who received services at the church had been apprehended by CBP and had been released from custody pending their court date, with an average length of time between arrival at the border and transfer to the church (i.e., length of time in ICE custody) of 3.0 days (SD = 2.1 days; range: 0 to 17). Families spent varying amounts of time at the church, ranging from less than one hour to approximately ten hours. Data were collected between August and December of 2014, but often for only portions of each day (most days). Although the church provided services to children as well as adults, only those individuals over 18 years of age were included in these analyses. The vast majority of individuals who were approached provided informed consent to be interviewed, however it was not possible to determine the proportion of individuals who declined to be interviewed (e.g., some research personnel did not record refusals and many individuals cited time constraints as the basis for not participating).

In total, 234 adults participated in the study; 114 from El Salvador (48.7%), 74 from Honduras (31.6%), and 46 from Guatemala (19.7%). The sample included 198 women (84.6%) and 36 men (15.4%), ranging in age from 18 to 62 years old (mean = 29.83, SD = 7.3). Most participants were married/partnered (*n* = 120, 51.3%) and described their economic status as “poor” (*n* = 165, 71.1%). Most participants (*n* = 133; 58.1%) had less than a high school education; 71 individuals reported having completed high school (31%), and 25 participants had completed a college education (10.9%).

### Procedures

Data were collected in private rooms at the church, using a structured interview developed by the study authors (S1 Table), and typically lasted 30 to 45 minutes. Following completion of the interview, participants were administered two self-report measures of psychological distress (described below), both of which had been previously translated into Spanish. Prospective participants were informed of nature of the study, that their involvement was entirely voluntary, and that choosing not to participate would have no effects on the services they received at the church. Study interviewers (*n* = 8) included medical doctors, psychologists, lawyers, and nuns, all of whom had previous experience working with an immigrant population. Interviewers were either bilingual (*n* = 7) or worked with the assistance of a Spanish-speaking interpreter (*n* = 1). Some participants were unable to complete the entire study, typically because they had scheduled transportation to meet family members living elsewhere in the U.S.

A structured interview was developed to gather information about pre-migration exposure to traumatic events and acts of persecution, the extent to which the individual had access to legal recourse or governmental protection from violence and persecution, whether the individual was afraid to return to their home country. After pilot testing, the final version of the interview consisted of 41 questions, including demographic characteristics, reasons for leaving the home country, trauma and persecution events experienced in the home country, and experiences related to the journey to the U.S. and arrival at the border. All interviews were conducted in Spanish. Participants were also informed that their responses to the study interview and questionnaires would have no bearing on their legal status or services provided by the church.

**Fig 1:**
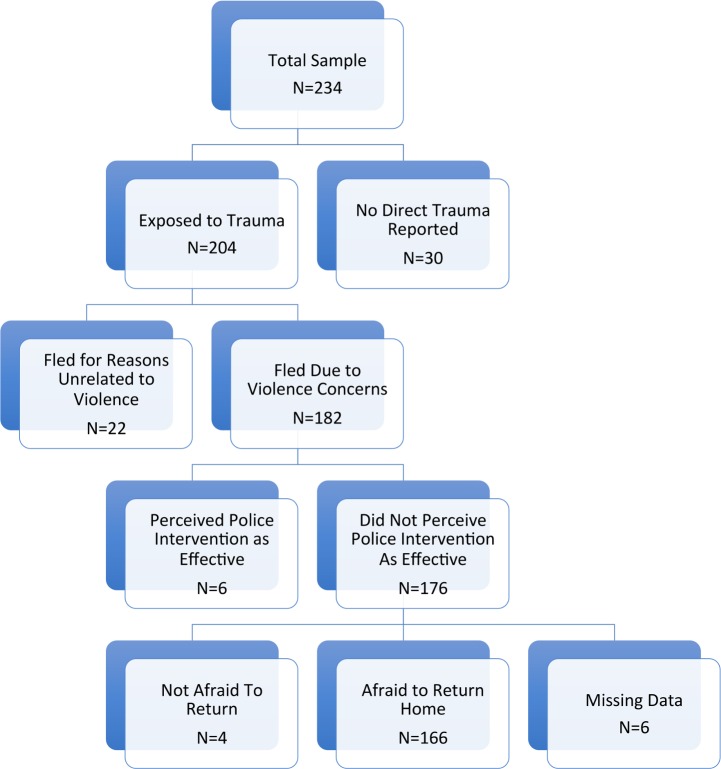
Determing Probable Asylum Status.

In order to ascertain whether migrants from the current sample described experiences that satisfied the criteria for consideration as an asylum-seeker, we developed an algorithm that mapped participant responses to the structured interview onto the core criteria for asylum (see Fig 1). This analysis included four components: (i) the person experienced threats or violence in their home country, (ii) the individual fled their home country due to violence and persecution, (iii) the individual did not perceive themselves as having access to effective legal recourse or protection in their home country, and (iv) the individual feared for their safety should they be forced to return to their home country. In order to meet the criteria for asylum seeking status, the participant had to endorse all four of these criteria.

Two measures of psychological distress were administered during this study, the Harvard Trauma Questionnaire (HTQ) [16] and the Patient Health Questionnaire-9 (PHQ-9). [17] Each of these instruments was administered orally (in Spanish) due to literacy concerns, using previously validated translations. The HTQ measures PTSD symptoms described in the Diagnostic and Statistical Manual of Mental Disorders (DSM-IV). [18] The HTQ was initially developed for use with Indochinese refugee populations and has since been translated into many languages and used with many cultures. [19–21] The PHQ-9 is a 9-item self-report measure assessing depressive symptoms as defined by the DSM-IV. This instrument has also been widely used in both the U.S. and international settings, including with Spanish-speaking samples. [22–24] Individuals that were identified as having serious mental health symptoms were provided with referral information on how to obtain mental health services at their destination.

Data analysis included descriptive statistics to characterize pre-migration trauma and rates of psychological symptoms, and frequency analyses to examine differences in rates of traumatic events across migrants from the three countries. Pearson product-moment correlation coefficients were used to quantify the association between psychological distress and length of time in ICE detention prior to release. This study was approved by the Institutional Review Board of the New York University Langone School of Medicine.

## Results

### Trauma Exposure

Table 1 presents reported traumatic experiences broken down by country of origin. Participants were asked about their exposure to a variety of traumatic events including violent acts, sexual violence, death threats, murder of family members, extortion, and kidnapping. Reports of serious violence were extremely common, with one third of participants (32.2%) reporting that a family member had been murdered. Death threats were also frequently reported, with roughly half of all participants reporting death threats towards themselves (45.4%) or their family (51.9%). Threats of violence (other than death) directed at themselves or a member of their family were even more common, with 57.8% reporting that they had received threats themselves and 66.2% reporting threats towards a family member. Other commonly reported traumatic experiences included extortion (33%) and domestic violence (29%; most, but not all of whom were women). Only 30 individuals (13%) reported no overt threats or violence towards themselves or a family member, although several of these individuals (*n* = 7) cited violence in their community as the primary reason for leaving their country. Overt threats and/or violence were significantly more common among migrants from El Salvador (92%) and Honduras (89%) compared to Guatemala (72%), Chi-Square = 12.55, df = 2, *p* = .002, phi = .23.

**Table 1:** Exposure to Violence Prior to Migration.

	El Salvador		Honduras		Guatemala	
	*n =* 115		*n* = 74		*n =* 46	
	Self	Family	Self	Family	Self	Family
Violence threats	79 (71.8%)	85 (77.3%)	34 (48.6%)	41 (60.3%)	16 (37.2%)	19 (46.3%)
Violence actual	7(6.5%)	50 (45.5%)	6 (8.5%)	34 (49.3%)	1 (2.2%)	9 (22.0%)
Death threats	61 (55.0%)	6 (61.0%)	29 (40.3%)	3 (51.5%)	13 (28.9%)	1 (30.2%)
Family murdered	N/A	38 (33.6%)	N/A	27 (37.0%)	N/A	9 (20.0%)
Sexual violence (threats)	10(9.2%)	9(8.1%)	9 (13.0%)	5(7.7%)	2(4.3%)	4 (9.1%)
Sexual violence (actual)	2(1.8%)	3(2.8%)	3(4.2%)	2(2.9%)	0	1 (2.4%)
Extortion	49 (45.0%)	32 (33.7%)	19 (26.8%)	13 (20.6%)	7 (15.9%)	4 (10.3%)
Kidnapping	1 (0.9%)	9 (8.2%)	1 (1.4%)	6 (8.7%)	0	0
Domestic violence	23 (21.9%)		28 (39.4%)		13 (30.2%)	

Although the rates of reported trauma were extraordinarily high among the sample as a whole, some traumatic experiences were more common among migrants from El Salvador. For example, 55% of participants from El Salvador reported receiving death threats towards themselves and 58% reported death threats towards a family member. The corresponding rate of death threats was 40% (towards themselves) and 51% (towards another family member) for those from Honduras and 29% (towards themselves) and 30% (towards family) for those from Guatemala; towards self: Chi-Square = 10.20, df = 2, *p* = .006, phi = .21; towards family: Chi-Square = 11.54, df = 2, *p* = .003, phi = .23. Physical violence towards a family member was also more common among those from El Salvador (45%) and Honduras (49%) compared to those from Guatemala (22%); Chi-Square = 8.78, df = 2, *p* = .012; phi = .20. On the other hand, rates of severe trauma were more comparable across countries, as the proportion reporting that a family member had been murdered was 34% among those from El Salvador, 37% from Honduras, and 20% from Guatemala; Chi-Square = 4.00, df = 2, *p* = .14, phi = .13. Likewise, rates of sexual assault were 2%, 4%, and 0% across the three countries (El Salvador, Honduras and Guatemala, respectively); Chi-Square = 2.36, df = 2, *p* = .31, phi = .10. Physical violence was also roughly comparable across groups, as 7% of participants from El Salvador reported experiencing physical violence themselves versus 8% of those from Honduras and 2% from Guatemala; Chi-Square = 1.84, df = 2, *p* = .40, phi = .09.

Participants were asked to describe the primary reason for fleeing their native country. The most common reason offered pertained to gang-related violence, reported by 138 individuals (60%). An additional 15 individuals cited domestic violence as their primary reason for fleeing (7%) and another 5 individuals cited other forms of violence (2%). However, when participants were asked about additional reasons for fleeing their country, another 33 individuals (14%) reported violence as the rationale. In total, 191 of 230 participants (83%; these data were missing for four individuals) indicated violence as a reason for fleeing their native country; of the 204 participants who reported experiencing threats or violence towards themselves or a family member, 182 (89%) cited violence as a reason for fleeing.

Most participants indicated that they did not report the problems they had experienced to the police, however of those who did (*n* = 59; 31%), the police response was typically perceived as ineffective or even detrimental (*n* = 47 of 53; 89%; perceived effectiveness of legal system interventions was missing for six individuals). Common reasons offered for not reporting violence to the police were fears of gang retaliation (*n =* 42; 39%), concerns about police corruption (*n =* 25; 23%), or belief that a police report would be ineffective or detrimental (*n =* 27; 25%). The vast majority of participants (90%) reported being afraid to return to their native country. Based on the asylum algorithm described above (see Fig 1), roughly 70% of all participants (*n* = 159) met criteria for asylum-seeking status, with significant differences in the rate across the three countries (El Salvador: 79%, Honduras: 74% and Guatamala: 41%); Chi-Square = 22.75, df = 2, *p* < .001; phi = .32.

### Severity of Mental Health Symptoms

Rates of depression and PTSD were also high among migrants from all three countries, with 32% of participants (*n* = 51 of 157 that completed the HTQ) reporting symptoms indicative of PTSD and 24% (*n* = 36 of 148 that completed the PHQ-9) for major depressive disorder. Of the 144 individuals that completed both the HTQ and PHQ-9, 25 (17%) reported symptoms that satisfied the criteria for both PTSD and depression whereas 85 (59%) had neither disorder. Rates of PTSD and depression were roughly comparable across the three countries, with PTSD rates of 32%, 34%, and 30% among migrants from El Salvador, Honduras, and Guatemala respectively; Chi-Square = 0.15, df = 2, *p* = .93; phi = .03. Rates of depression were 27%, 29% and 8%, among individuals from El Salvador, Honduras and Guatemala; Chi-Square = 4.04, df = 2, *p* = .13, phi = .17. There was no association between length of time in ICE detention and severity of psychological distress (HTQ: *r* = .04, *p* = .67; PHQ-9: *r* = .11, *p* = .24).

## Discussion

These data provide a stark contrast to the description of Central American immigrants contained in many media reports and political debates, as migrants motivated primarily by economic incentives. Structured interviews revealed high rates of trauma exposure, often including murdered family members, sexual and physical assault, death threats, extortion and kidnapping. Very few participants reported that notifying the authorities resulted in an improvement in their situation though most respondents acknowledged that they did not even try to seek assistance for fear of retaliation. The vast majority of those interviewed expressed a fear for their safety if forced to return to their native country. In short, more than two thirds of those interviewed described experiences and conditions that, if accurate, would satisfy the requirements for asylum in the U.S. and many other countries. [25] This conclusion rests on the assumption that persecution by a non-governmental group (i.e., “gangs”) can form the basis for an asylum claim, an argument that has been increasingly accepted by courts in the U.S. and other developed countries. [26–27]

Although rates of trauma were high across all three subgroups of migrants, there were national differences in the rates of many trauma experiences. Significantly more migrants from El Salvador and Honduras described physical violence and threats, and their experiences were more likely to satisfy the legal requirements for asylum. These findings should not be taken as a dismissal of the trauma experienced by refugees from Guatemala, as nearly half of those individuals were exposed to severe trauma, perceived no legal recourse from the authorities and feared for their safety if forced to return. Moreover, the relatively lower rate of trauma reported by Guatemalan migrants may reflect greater difficulty escaping from the violence in this region. Without further information on the conditions in each of these countries, this possible explanation cannot be examined.

The severity of psychological distress among study participants was also striking, as roughly one third of the participants endorsed clinically significant PTSD symptoms and one quarter endorsed symptoms consistent with a major depressive disorder. Although the cross-sectional methodology used in this study prevents any firm conclusions regarding the etiology of reported psychological symptoms, the association between political violence and psychological symptoms is well established. [28–30] There was, however, no association with length of time in ICE detention, likely due to the modest length of time participants had been detained (a mean of three days). Moreover, the rates of psychological distress observed in this study undoubtedly underestimate the true prevalence of psychiatric diagnoses due to the limited range of symptoms assessed. We focused only on symptoms of PTSD and depression, though symptoms of anxiety and somatization are also quite common among refugees and trauma-exposed individuals. [29–30]

One caveat in this analysis is the difficulty determining whether any legal recourse was available within the participants’ native country. Only a small subset (25%) of participants had reported the threats or violence they experienced to the authorities and only six of these individuals described police intervention as effective (11% of those that reported the violence to the police). Rather, most participants indicated that they did not make a formal report, as they anticipated that this would either be ineffective or might even worsen the situation. It is not possible to determine whether the perceptions of study participants–that no effective recourse through existing legal channels was available to them–are accurate, but the low rate of effective intervention among the subset that did make reports to the authorities provides some support for this perception.

There were a number of additional limitations to the study that should also be considered. First, the potential for distorted responding always exists, particularly given that all participants individuals were seeking entry into the U.S. Although these individuals were informed that their responses were confidential and would not impact their legal status, it is certainly possible that some participants deliberately fabricated or exaggerated the nature of their experiences. However, the relatively low frequency of many of the more “serious” traumatic events, such as physical or sexual assault, suggests that deliberate exaggeration or fabrication of trauma exposure was unlikely. The discrepancies observed across the three countries also support the credibility of these reports, as the three samples had significantly different rates of trauma exposure (presumably reflective of the relative frequency of violence across the three countries). Further, while rates of depression and PTSD were far higher than those found in typical community samples, they were roughly comparable to the rates observed in other studies of trauma-exposed refugees, including samples that had no discernible incentive to exaggerate psychological symptoms. In short, while the veracity of participant reports cannot be assured, these indicators support the credibility of these reports.

Another important limitation pertains to our analysis of the criteria for asylum. We based the algorithm used in this study on our review of the legal standards, but the extent to which this approach accurately captures these legal standards is largely unknown. Specifically, we did not have the opportunity to “validate” this algorithm by comparing the classification results to actual asylum determinations. Thus, these data are best characterized as an approximation of asylum criteria, rather than a legal standard *per se*. Finally, the extent to which this sample is representative of all individuals and families migrating to the U.S. from the Northern Triangle region is unknown. Although we are unaware of any systematic basis for transporting families through the study site, the potential for systematic bias in the sample exists.

These caveats notwithstanding, these data demonstrate high rates of trauma and psychological distress, and suggest that roughly two thirds of individuals from the Northern Triangle region of El Salvador, Honduras and Guatemala might meet the legal requirements for asylum in the U.S. Although some discrepancies in this rate were observed across the three countries, the vast majority of migrants from El Salvador and Honduras satisfied these requirements, as did nearly half of the migrants from Guatemala. Coupled with the high rates of severe psychological symptoms, there is little doubt that immigration policies such as the frequent use of detention need to be reconsidered.

## Supporting Information

S1 Table Final Survey for Central America. (DOCX)
