# Supplementary material for: Pre-Migration Trauma Exposure and Mental Health Functioning among Central American Migrants Arriving at the US Border
Source: PLoS One. 2017 Jan 10;12(1):e0168692. doi: 10.1371/journal.pone.0168692 (PMC5224987; doi:10.1371/journal.pone.0168692)
Supplement: S1 Table — (DOCX) [file pone.0168692.s001.docx]

I confirm that Informed Consent was obtained (interviewer initials) _______________

*Start Time: *End Time:

1. Gender M F
2. Age
3. Country ________________ City ______________
4. Marital status: Single Married/Partnered Divorced Widowed

5. Level of education: a. No formal education b. Elementary school c. Middle School

d. High School e. College

1. Read and write: Yes No

7. Occupation:

8. Socioeconomic class: Poor Middle Wealthy

9. Family members travelling with client: (a) Relationship: Age: _____

(b) Relationship: Age: _____ (c) Relationship: Age: _____

(d) Relationship: Age: _____

10. Family remaining in home country? a. Yes b. No

10.A. a. Spouse b. Children <18 c. Parents/sibling d. Other family members

11. Family in USA to be re-united with: a. Yes: City: State: _____ b. No

11.A. a. Spouse b. Children <18 c. Parents/sibling d. Other family members

12.A. Why did you leave your country? In other words, what is the main reason you left your country?

i.

12.B. Are there other reasons for why you left your country ? ***(Rank order them)***

ii.

iii.

13. Reason for leaving country at this particular time (instead of sooner or later):

14. A. Was violence an important factor in your decision to leave? Yes No

B. Was poor economic condition an important factor in your decision to leave? Yes No

*(Explain, including why one is more or less than other)*

15. If there was no violence would you still have left? a. Yes b. No

Explain:

**In your country, have you or a family member been:**

16. **Threatened with violence?**

1. Self i. Yes ii. No
2. Family Member i. Yes ii. No *Specify: ______________________________________*

If yes, by whom *(check appropriate boxes)*

☐ Gang Members ☐Police ☐Other Govt./Military ☐Family member

☐ Other *Specify: ______________________________________*

When: *(Month/year)*

Describe all incidents:

17. **Experienced physical violence?**

1. Self i. Yes ii. No
2. Family Member i. Yes ii. No *Specify: ______________________________________*

If yes, by whom *(check appropriate boxes)*

☐ Gang Members ☐Police ☐Other Govt./Military ☐Family member

☐Other *Specify: ______________________________________*

When: *(Month/year)*

Describe all incidents:

18.  **Received death threats?**

1. Self i. Yes ii. No
2. Family Member i. Yes ii. No *Specify: ______________________________________*

If yes, by whom *(check appropriate boxes)*

☐ Gang Members ☐Police ☐Other Govt./Military ☐Family member

☐ Other *Specify: ______________________________________*

When: *(Month/year)*

Describe all incidents:

19. **Has anyone in your family been murdered?**

i. Yes ii. No *Specify: ______________________________________*

If yes, by whom *(check appropriate boxes)*

☐ Gang Members ☐Police ☐Other Govt./Military ☐Family member

☐ Other *Specify: ______________________________________*

When: *(Month/year)*

Describe all incidents:

20. **Threatened with sexual violence?**

1. Self i. Yes ii. No
2. Family Member i. Yes ii. No *Specify: ______________________________________*

If yes, by whom *(check appropriate boxes)*

☐ Gang Members ☐Police ☐Other Govt./Military ☐Family member

☐Other *Specify: ______________________________________*

When: *(Month/year)*

Describe all incidents:

21. **Experienced sexual violence?**

1. Self i. Yes ii. No
2. Family Member i. Yes ii. No *Specify: ______________________________________*

If yes, by whom *(check appropriate boxes)*

☐ Gang Members ☐Police ☐Other Govt./Military ☐Family member

☐ Other *Specify: ______________________________________*

When: *(Month/year)*

Describe all incidents:

22. **Forced to pay someone or do something because you were afraid?**

1. Self i. Yes ii. No
2. Family Member i. Yes ii. No *Specify: ______________________________________*

If yes, by whom *(check appropriate boxes)*

☐ Gang Members ☐Police ☐Other Govt./Military ☐Family member

☐ Other *Specify: ______________________________________*

When: *(Month/year)*

Describe all incidents:

23. **Been kidnapped?**

1. Self i. Yes ii. No
2. Family Member i. Yes ii. No *Specify: ______________________________________*

If yes, by whom *(check appropriate boxes)*

☐ Gang Members ☐Police ☐Other Govt./Military ☐Family member

☐ Other *Specify: ______________________________________*

When: *(Month/year)*

Describe all incidents:

24. Have you or your children **experienced abuse or violence in your family?**

*(NOTE: THIS REFERS TO FAMILY VIOLENCE, EG. SPOUSAL ABUSE, ETC)*

☐ Yes ☐ No

If yes, by whom and when?

Describe:

25. *(If yes to 16-24)* Regarding the experiences you just told me about: **Did you report any of them to officials, such as the police, in your country for help?** ☐Yes ☐No

If yes, specify who you asked and what happened?

If no, why didn’t you ask for help?

26. **Threatened by government officials such as the police or military?**

1. Self i. Yes ii. No
2. Family Member i. Yes ii. No *Specify: ______________________________________*

If yes, by whom *(check appropriate boxes)*

☐Police ☐ Military ☐ Other Govt. officials *Specify: ______________________________________*

When: *(Month/year)*

Describe all incidents:

27. **Unfairly arrested, imprisoned, beaten or tortured by police or other government authorities?**

1. Self i. Yes ii. No
2. Family Member i. Yes ii. No *Specify: ______________________________________*

If yes, by whom *(check appropriate boxes)*

☐Police ☐ Military ☐ Other Govt. officials *Specify: ______________________________________*

When: *(Month/year)*

Describe all incidents:

28. i. *(if applicable)* **Do you or your children who came with you have any health problems as a result of the experiences you described to me?**

a. Yes b. No

If yes, explain

ii. Physical Health Problems?

a. Yes b. No

If yes, explain

iii. Emotional Health Problems?

a. Yes b. No

If yes, explain

29. **Afraid for the safety and well-being of your family members your country?**

a. Yes b. No

If yes, why (specify, who what, and where):

30. **Are you afraid to return to your country?** a. Yes b. No

If yes, why?

*(if interviewee does not mention “life in danger”, then specifically ask if they think their life will be in danger if they return to their country)*

☐Life in Danger

☐Other (specify) ____________________

**Journey to the United States:**

31. Date of departure from home country:

32. i. Threats of violence during journey: a. Yes b. No

If yes, explain:

ii. Victim of violence during journey: a. Yes b. No

If yes, explain:

iii. Fear for your life: a. Yes b. No

If yes, explain:

**US border Crossing:**

33. Date of arrival at US border:

34. Date of arrival at Sacred Heart:

35. i. Did US immigration officials ask if you are afraid to return to your country?

a. Yes b. No

If yes, what did you say? What was their response?

***If no to 35i. ask 35ii:***

ii. Did U.S. Immigration officials ask if you are afraid because of your children to return to your country?

a. Yes b. No

If yes, what did you say? What was their response?

***If no to 35i. and 35ii. Skip to question 36***

36 . i. When you were asked if you are afraid to return to your country, was it in a private place? a. Yes b. No

If no, describe:

ii. When you were asked if you are afraid to return to your country, were your children with you?

a. Yes b. No

iii. If yes, did this make it more difficult for you to answer the question about whether you are afraid to return to your country?

a. Yes b. No

(Explain)

37. i. Did U.S. Immigration officials accuse you of lying?

a. Yes b. No

If yes, explain:

ii. Did U.S. Immigration officials say anything to discourage you or others from coming to the U.S.?

a. Yes b. No

If yes, explain:

38. Treated fairly by US Immigration officials? a. Yes b. No

Describe:

39. Were conditions under which you were held at the border adequate? a. Yes b. No

Explain:

40. If conditions improve in your country, do you want to return home? a. Yes b. No

Why or why not?

41. Do you feel you have been treated well here at Sacred Heart? a. Yes b. No

Explain:
